# “We’ve managed to find our own path of how we do it”: exploring parental perceptions of using age-inappropriate formulations at home—a qualitative study

**DOI:** 10.3389/fped.2026.1705984

**Published:** 2026-03-09

**Authors:** Jennifer C. Duncan, Louise E. Bracken, Daniel B. Hawcutt, Matthew Peak, Mark A. Turner

**Affiliations:** 1 Department of Women’s & Children’s Health, Institute of Life Course and Medical Sciences, University of Liverpool, Liverpool, United Kingdom; 2 Paediatric Medicines Research Unit, Alder Hey Children’s NHS Foundation Trust, Liverpool, United Kingdom

**Keywords:** children, home, medicines administration, off-label medicines, parent, qualitative research, theoretical framework of children's medicine acceptability, unlicensed medicines

## Abstract

**Introduction:**

The administration of medicines to children poses distinct challenges, largely due to the limited availability of authorised, commercially available, age-appropriate formulations (AaFs). In the absence of suitable options, parents and carers are often required to manipulate age-inappropriate formulations (AiFs) to facilitate administration. However, little is known about their perceptions and experiences when administering AiFs to children at home.

**Methods:**

Qualitative exploratory study. Participants were recruited from a UK paediatric hospital. Semi-structured interviews were conducted either in participants’ homes or at the hospital. Interviews were audio-recorded, transcribed verbatim, and thematically analysed using NVivo v10. Topics explored included formulation types, dosage form manipulation, medicine supply, and administration practices.

**Results:**

Over sixty outpatient clinics were screened; 34 families expressed interest, and 13 completed interviews between September 2016 to February 2017 (mean duration: 35 min). Eleven interviews were conducted at home and two in hospital; 12 participants were mothers, and 8 of the 13 children discussed were female. Thematic analysis identified five key themes: (1) inappropriate formulations; (2) unlicensed medicine and off-label prescribing; (3) framework of appropriateness; (4) knowledge, training, communication, and relationships; and (5) patient and family experience. Parents often relied on prescriber instructions without fully understanding the implications of manipulating medicines, indicating a gap in support and knowledge transfer.

**Discussion:**

These findings highlight the emotional and practical burdens families face when administering age-inappropriate medicines, including the time and effort required to ensure accurate dosing and safe administration. The study emphasises the need for increased access to child-appropriate formulations of essential medicines and more child-centred prescribing practices. Where legacy medicines remain the only option, healthcare professionals can support children to safely swallow solid oral dosage forms, such as tablets, and educate families on safe medicine manipulation. Further research is needed to better understand the magnitude of these burdens and guide future paediatric formulation development.

## 1 Introduction

There is a well-recognised lack of age-appropriate pharmaceutical formulations available on the market for paediatric use (1–3), leaving children at risk of unsafe or ineffective treatment. Well-designed medicines improve safety, offer an enhanced “user” experience and support adherence to treatment in children (4, 5). Like adults, children deserve access to high-quality, licensed medicines which have been thoroughly researched and tailored to meet their specific needs. However, developing new, age-appropriate medicines, designed with children in mind requires substantial financial investment and time. The paediatric population is small, yet hugely diverse, due to the developmental changes and physiological differences which occur across the stages of childhood (6–8). In addition, particularly for younger children, medication is typically administered by a parent or caregiver in the home setting. This presents a unique challenge when developing, prescribing, and administering medicines to children (9).

To meet individual patient needs, medicines are often prescribed in paediatrics outside the terms of their marketing authorisation (MA), referred to as “off-label” (OL), or involve the use of unlicensed medicines (UL), which may lack regulatory approval in the UK or globally (10–15). Consequently, modification (manipulation or handling) of available medicines at the point of use by carers to achieve the “appropriate” dose (often smaller) and/or to allow successful administration of the medicine to a child has become standard practice (16–18). Once the child is discharged from hospital, the responsibility for carrying out these manipulations as part of the medicine administration process shifts to the parents and carers. While healthcare professionals (HCPs) may view this as “business as usual,” it is unclear whether carers fully grasp the implications of performing these manipulations. Such practices may create additional barriers to the safe and effective administration of medicines, increasing the risk of medication errors and potentially compromising children's safety at home.

The challenges of administering age-inappropriate formulations (AiFs) are not only technical but are also shaped by broader family and socio-cultural factors. Parents' ability to give medicines safely is influenced by time pressures, stress, and daily responsibilities, which can limit their capacity to follow instructions or adapt adult medicines for children (19). In many families, cultural expectations influence who provides care, and a child's gender can affect how much attention and support they receive, which may in turn shape medicine administration at home (20). Together, these factors show that managing age-inappropriate medicines goes beyond practical skills, highlighting the importance of understanding parents' experiences to improve safety and support.

This qualitative exploratory study therefore aimed to explore these issues by examining the experiences of parents and carers responsible for administering AiFs to children in the home environment, addressing a critical gap in understanding. The goal was to gain deeper understanding the practical and emotional challenges this practice may present. Parents of children who were prescribed an AiF, typically intended for use in adults, were interviewed to gather real-life user experience data. Reasons for the use of AiFs as part of routine clinical care included availability, cost of “unlicensed specials” or patient or parental preference.

## 2 Methods

**Figure 1:**
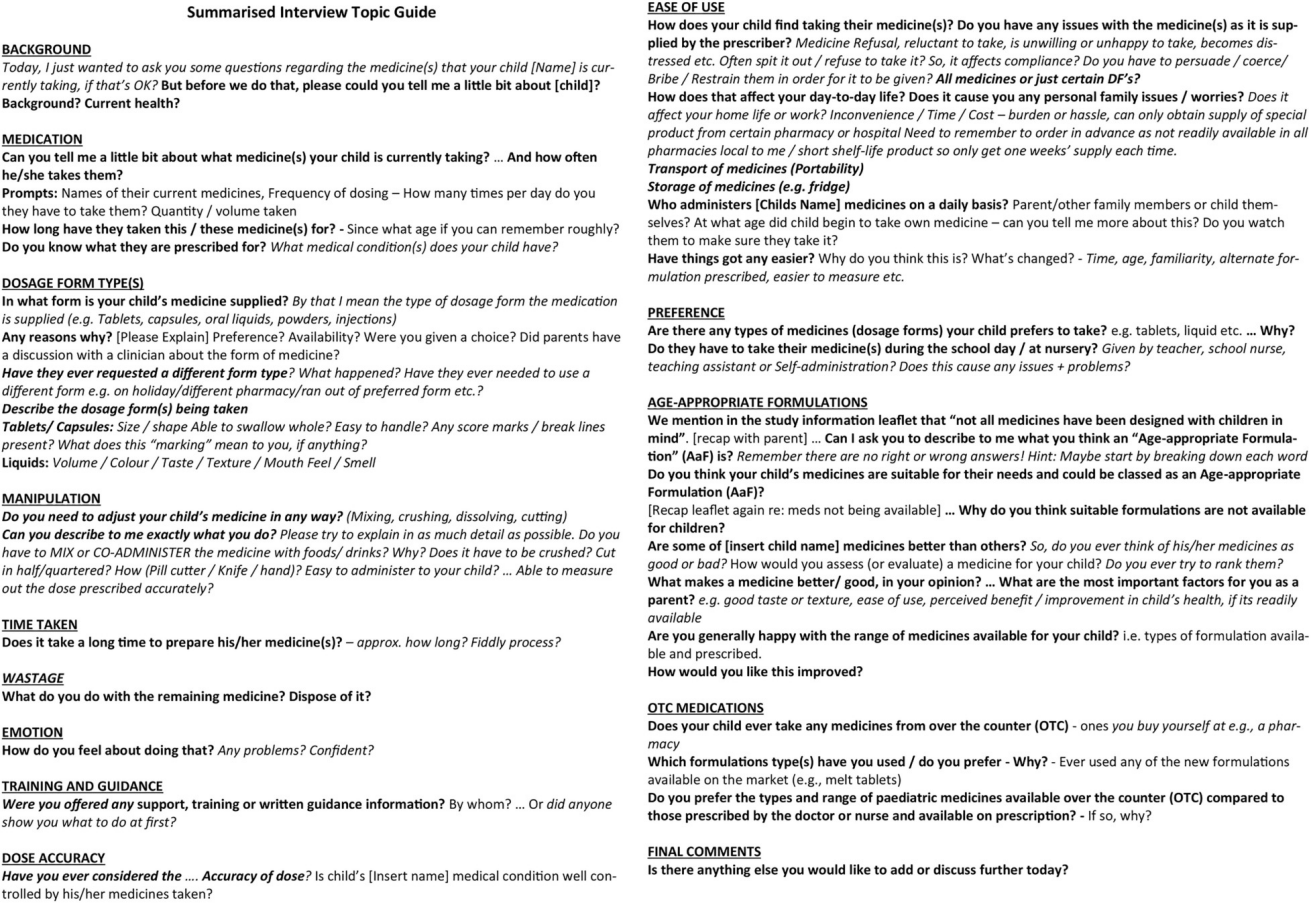
Summarised interview topic guide.

A qualitative exploratory study using individual semi-structured interviews was conducted to explore the experiences of parents and carers responsible for administering and manipulating AiFs to children at home. No *a priori* theory or conceptual framework informed the study design. All study procedures were conducted in accordance with ethical standards and regulatory requirements. Thematic analysis was chosen to identify central themes emerging from the dataset and to structure the data meaningfully (21, 22). Themes were interpreted by examining common relationships, patterns, theoretical constructs, and explanatory principles (23, 24). Data were generated using the study interview topic guide (Figure 1).

### 2.1 Setting and participants

Eligible participants were parents of children aged 1–15 years who were currently taking one or more AiFs, as defined using the AaF Assessment Tool (25). This age range was chosen to capture a variety of developmental stages, recognising that parental experiences may differ between parents of younger children and teenagers. Purposive sampling targeted specialty areas where manipulated medicines are commonly prescribed to children for the management of long-term medical conditions (LTMCs). Participants were approached by JCD during their child's routine outpatient clinic appointment at Alder Hey Children's NHS Foundation Trust (AH). Recruitment was opportunistic, and participation was voluntary, based on parent availability and willingness to take part. Expressions of interest were recorded, and families were subsequently contacted to arrange interviews.

Participants were assigned a unique identifier (e.g., AiF-P001) at the point of expression of interest. Numbering reflects all initial expressions of interest rather than completed interviews and was used solely to maintain confidentiality. Eligibility criteria included a confirmed diagnosis, and that the child had been taking the identified age-inappropriate medicine(s) for a minimum of three months prior to the parental interview. Medicines could be administered via any route, including enteral tubes such as nasogastric tube (NGT) or percutaneous endoscopic gastrostomy (PEG). Non-English-speaking parents/carers were excluded. Recruitment was not restricted by specific medical diagnoses or medicine types to ensure a broad range of AiFs were captured. Demographic data such as parental education, socioeconomic status, and ethnicity were not collected, as this was outside the scope of the approved study protocol.

### 2.2 Sample size

Sample size was not predetermined. Consistent with Braun & Clarke's reflexive thematic analysis principles (23, 24) it was guided by the richness and depth of the data rather than statistical considerations. Recruitment and interviews continued until sufficient information power was achieved, enabling in-depth exploration of parental experiences across a range of medicines, ages, and administration routes (26). Later interviews indicated that no new themes emerged, supporting that sufficient information power had been reached.

### 2.3 Procedure and reflexivity

The study was conducted in accordance with Consolidated Criteria for Reporting Qualitative Research (COREQ) guidelines (27). All interviews were carried out by JCD, an experienced healthcare professional (Research Pharmacist) trained in qualitative research methods. Interviews were conducted on a single occasion, either in the hospital or at the participants' homes, and were audio recorded. Written informed consent was obtained immediately prior to each interview. Non-participants [e.g., child/sibling(s)/partner] were occasionally also present during the interview, however only participant remarks were included in the analysis. A semi-structured interview approach allowed participants to describe their experiences in their own words and to discuss unanticipated topics. The topic guide was iteratively refined throughout the study as data collection progressed. Field notes were recorded by JCD after each interview, including details of medication brand and manufacturer. A pilot interview did not take place. Transcripts were not returned to participants for comment, and participant feedback on findings was not sought.

Reflexivity was considered throughout the study, in line with Braun & Clarke's reflexive thematic analysis approach, which recognises researcher subjectivity as integral to knowledge production (23, 24, 28, 29). The interviewer's expertise in paediatric medicines and pharmacy helped them interpret participants' descriptions accurately and probe for relevant details. To minimise potential bias, participants were reminded that there were no “right” or “wrong” answers. Reflexive discussions within the multidisciplinary research team—including a nurse, doctor, scientist/biochemist, and pharmacists with varying experience—were conducted during coding and analysis to support balanced interpretation and ensure findings reflected participants' perspectives.

### 2.4 Data analysis

Interviews were transcribed verbatim, checked for accuracy and anonymised to remove all identifiable information. NVIVO Version 10 (QSR International, 2012) was used soley to support data organisation. Theme development and coding decisions were guided by iterative review of the transcripts and collaborative discussion among the research team (22–24).

Familiarisation and iterative examination of the transcripts ensured immersion and understanding (22, 24). Data were coded inductively into categories and subcategories, allowing patterns and themes to emerge from the dataset (22, 24). Coding was conducted collaboratively by two researchers, with initial codes discussed and refined to enhance analytic depth and reflexivity (23, 24).

Themes and categories were iteratively refined through discussion within the multidisciplinary research team, resulting in a robust analytical framework reflecting participant's perspectives. Coded data were reviewed and interpreted both within individual interviews and across the dataset. Analytical decisions and theme development were discussed to enhance reflexivity, transparency, and consistency of interpretation.

Quotations were selected to illustrate key themes and a range of participant experiences, prioritising clarity, relevance, and representation of both typical and less common perspectives.

## 3 Results

JCD attended more than 60 outpatient clinics, from which expressions of interest and contact information were obtained from 34 families. Reasons for non-participation included being uncontactable during the recruitment period (*n* = 7), not meeting study eligibility criteria following medicine review (*n* = 6), declining to participate (*n* = 5), repeated cancellations (*n* = 2), and ineligibility on clinician advice (*n* = 1). Participants are referred to using unique identifiers (e.g., AiF-P001), with numbering up to AiF-P034 reflecting all expressions of interest rather than the number of completed interviews.

Thirteen interviews were conducted over a six-month period (September 2016 to February 2017). Eleven were carried out in the participants' homes, and the remaining two took place in a private room at the hospital. The interviews ranged in length from 15 to 64 minutes (mean: 35 min). The patient families (*n* = 13) were recruited from the following clinical specialties: Cardiology (*n* = 8), Nephrology (*n* = 3), Endocrinology (*n* = 1), and Respiratory (*n* = 1). The median age of the children discussed as part of the interviews was three years (range: 1-13). Eight of the 13 (61.5%) children were female. Only one interview was conducted with the child's father, all others were with the child's mother (Table 1).

**Table 1:** Details of interviews including child demographic.

Parent ID (AiF-)	Interview location	Length of Interview (HH:MM:SS)	Child age	Child gender	Relationship to child	Clinical team	Child diagnosis
P003	Hospital	00:15:04	2	F	Mother	N	Kidney failure
P005	Hospital	00:46:27	3	M	Mother	N	Heart failure—cardiomyopathy, Renal artery stenosis (bilateral), High blood pressure
P006	Home	00:36:00	13	F	Mother	N	Post-kidney transplant
P007	Home	00:18:55	13	F	Mother	C	Supraventricular Tachycardia (SVT)
P009	Home	00:40:21	4	M	Mother	R	Cystic fibrosis (CF)
P016	Home	00:33:54	1	F	Father	E	Congenital adrenal hyperplasia (CAH)
P021	Home	00:15:55	3	F	Mother	C	Complete atrioventricular septal defect (AVSD)
P025	Home	00:40:08	2	F	Mother	C	Stroke, Patent ductus arteriosus (PDA)
P026	Home	00:25:53	11	M	Mother	C	Joint hypermobility, unable to regulate blood pressure
P027	Home	00:26:50	3	M	Mother	C	Hypoplastic right heart, Transposition of great arteries (TGA), pulmonary stenosis
P032	Home	00:43:06	4	F	Mother	C	Truncus arteriosus, Autism, Sleep apnoea, Reflux
P033	Home	00:51:34	1	M	Mother	C	Hypoplastic left heart
P034	Home	01:04:56	8	F	Mother	C	Williams syndrome, post-heat transplant, learning difficulties

F, Female; M, Male; C, Cardiology; R, Respiratory; E, Endocrine; N, Renal (Nephrology).

The list of AiFs discussed as part of the interviews includes details of the manipulation/modification which occurred and the brand/manufacturer of the prescribed medicine (Table 2). During the interview parents also mentioned their child's concurrent medication(s) and sometimes recalled medicines which were no longer prescribed, commenting on historical issues or previous memorable events.

**Table 2:** AiF medicines discussed as part of interviews.

Parent ID (AiF-)	Child age	TARGET AiF(s)	Product licence status	Brand/manufacturer	Required dose	% of original dose	Frequency	Route	Manipulation details
P003	2	Aspirin 75 mg disp tablet	OLAM	M & A Pharmachem Ltd	37.5mg	50%	OD	NGT	FULL tablet dispersed in 5 mL of water and given 2.5 mL [Proportional dose]
		Levothyroxine 25 mg tablet	OLAM	Wockhardt UK Ltd	25mg	100%	OD		FULL tablet, dispersed in water (volume not stated)
P005	3	Aspirin 75 mg disp tablet	OLAM	M & A Pharmachem Ltd	37.5mg	50%	OD	PEG	HALF a tablet, dispersed in water (approx 2.5 mL) [Proportional dose]
		Losartan 25 mg tablet	OLAM	Accord Healthcare Limited	25mg	100%	OD		FULL tablet, dispersed in water (approx 5–10 mL)
		Lansoprazole 15 mg MUPS	OLAM	Zoton FasTab® (Pfizer Ltd)	7.5mg	50%	OD		HALF a tablet, dispersed in water (approx 5–10 mL) [Proportional dose]
P006	13	Trimethoprim 100 mg tablet	OLAM	Variable - Crescent Pharma Ltd (Scored)/Kent Pharmaceuticals (Non-scored)	50mg	50%	OD	Oral	HALF a tablet swallowed whole [Proportional dose]. Purpose of break-line not stated on Crescent SmPC but prophylaxic dose for children suggested is from 50 mg. With Kent pharmaceuticals product, no break-line on this brand. Both AiF=As per AaF assessment guide.
P007	3	Flecainide 50 mg tablet	OLAM	Actavis UK Ltd	75 mg	150%	BD	Oral	Tablet cut in HALF a tablet, then 1.5 tablets swallowed whole [Proportional dose]
P009	4	Vitamin K 10 mg tablet (Menadiol)	OLAM	Alliance Pharmaceuticals Ltd	5 mg^a^	50%	OD	Oral	HALF a tablet being dispersed in water (= AiF) [Proportional dose].^b^
P016	1	Fludrocortisone 100 mcg tablet	OLAM	Aspen Pharma	75 mcg	75%	OD	Oral	FULL tablet cut in HALF, then one half cut into QUARTERS. THREE QUARTERS of a tablet, dispersed in juice [Proportional dose]
P021	3	Lisinopril 1 mg/mL oral solution	OLAM	Essential Pharmaceuticals	1.25mg	N/A	OD	Oral	1.25 mL mixed with chocolate milk [Palatability issue]
P025	2	Aspirin 75 mg disp tablet	OLAM	Variable - Aspar Pharmaceuticals Ltd or Bristol Laboratories Limited	37.5mg	50%	OD	Oral	HALF a tablet dispersed in water, previously been on FULL tablet [Proportional dose]
P026	11	Fludrocortisone 100mcg tablet	OLAM	Aspen Pharma	50mcg	50%	OD	Oral	HALF a tablet, swallowed whole [Proportional dose]
P027	3	Aspirin 75 mg disp tablet	OLAM	M & A Pharmachem Ltd	37.5mg	50%	OD	PEG	HALF a tablet, dispersed in juice/water [Proportional dose]
P032	4	Melatonin 2 mg capsule	UL	Quantum Pharmaceuticals	4mg	200%	ON	Oral	TWO capsules opened and contents is mixed with drink
		Melatonin 2 mg PR tablet	OLAM	Circadin® (Flynn Pharma Ltd)	4mg	200%	ON		TWO prolonged release tablets being chewed as unable to swallow.
		Lansoprazole 15 mg orodispersible tablet	OLAM	Generics [UK] t/a Mylan	15mg	100%	OD		Full tablet dispersed in water (authorised) and mixed with juice to flavour (unauthorised)
P033	1	Aspirin 75 mg disp tablet	OLAM	M & A Pharmachem Ltd	37.5mg	50%	OD	Oral	HALF a tablet dispersed in water, previously been on QUARTER tablet [Proportional dose]
P034	8	Pravastatin 10 mg tablet	OLAM	Milpharm Limited	10mg	100%	OD	Oral	FULL tablet crushed whole (using spoons) and dispersed in water
		Prednisolone 5 mg soluble tablet	OLAM	Amdipharm UK Limited (Under Concordia International)	3mg	60%	OD		FULL tablet dispersed in 2.5 mL of water and given 1.5 mL [Proportional dose]

Disp, dispersible; MoA, Method of administration; OLAM, off-label use of authorised medicine; UL, Unlicensed special; OD, once daily; ON, at night; BD, twice daily.

^a^Dose now authorised, original MoA not.

^b^Child just recently started to swallow, not known until during interview (= AaF). SmPC was updated in June 2018 for this product and now states “the tablet can be divided into equal doses”, previously purpose of break-line was not specified.

Illustrative quotations are used throughout to support the findings. Five main themes were identified from the data: (1) inappropriate formulations; (2) unlicensed medicine and off-label prescribing; (3) framework of appropriateness; (4) knowledge, training, communication, and relationships; (5) patient and family experience. These key themes are summarised in Table 3 as part of the coding framework. Additional illustrative quotes are presented in Tables 4–8. Each theme is discussed further below.

**Table 3:** Key themes identified from the coding framework.

Key theme	Initial themes/open codes
Inappropriate formulations	DF related issues—organoleptic (e.g., taste/texture/smell) v physical (e.g., size/shape/volume) properties of the medicine, drug indication, stability, manufacturer differences, administration devices and equipment, comparison of DF types. Manipulation—description and type, consequence, safety concerns and risks, error, inaccuracy, drug wastage, mess, time factor, handling, drug efficacy, equipment, difficulty level, problems with “authorised” manipulations, past experiences. Views and perceptions—observations made during manipulations, disutility (inconvenience, hassle, harm, dissatisfaction, annoyance factor), acceptance of having to use DF supplied (no alternative), acknowledgement of inappropriate/suboptimal use.
Unlicensed medicine and off-label prescribing	ULOL use—associated risks, lack of guidance, wide selection of products causing confusion, supply issues, risk of switching brand/suppliers, lack of awareness, need for open conversation. Cost—prohibitive cost of liquid ’specials’ leading to inappropriate prescribing, affordability for NHS.
Framework of appropriateness	Value of improved formulation—Utility (effectiveness, convenience, value, and benefit), definition/knowledge of AaF, personalised medicine/individualised care, options, preference, what parents would like and what could be improved?
Knowledge, training, communication, and relationships	Relationship with HCPs—refusal to supply, challenged relationships, trust in HCPs, policy, and procedures. Knowledge and training—of disease and medicines, information source, variability, learning curve, lack of understanding, awareness of their actions, provides assurance/confidence, knowledge empowerment, share with others. Information & communication—lack of information/inconsistency, primary and secondary care interface, patient/family involvement in treatment options.
Patient and family experience	Compliance and adherence—Does supply of inappropriate formulation affect compliance to medication regime? consequence of inappropriate treatment, child obedience/behaviours. Impact on Family—concerns, hassle, feelings, inconvenience, stress and anxiety, patient access, availability and supply, time, responsibility, confidence, travel, patient related factors, peer pressure, dependence on carer/caregiver, complex dose regimes, environment (home/school/clinical setting). Challenges with administration and management—Challenges/barriers faced, route and method used, daily-life interruptions and unexpected events, interferes with family/social life, adapt life around it, obtaining further supplies and delays, travel, logistics, storage issues, ease of use, posology (ease of and change of dose), swallowability, number/frequency of medicines taken. Strategy development—Skills, methods and interventions needed to overcome issues faced by parents on administration e.g., developed own ‘improved’ method such as co-administration with food/drink or taking personal control. Familiarity—Becoming complacent over time as get familiar with process/medicines (just part of daily routine), learning from previous experiences

**Table 4:** Theme: inappropriate formulations additional participant quotations.

Key theme	Additional quotations
Inappropriate formulations	• “The medicine might be appropriate for him, but the formulation isn't because of the way that it's in a tablet and then we’ve got to cut it down, make sure it's dispersed in water and give it through his gastrostomy.” AiF-P027 [ADULT DFs] • “It's just like, the tablet itself is tiny and you have to try cut that in half.” AiF-P026 [HANDLING] • “It's more awkward you know chopping the tablet down than just obviously syringing it from a bottle into her juice.” AiF-P016 [HANDLING] • “It never breaks evenly right the way through so you know I wouldn't say that's a definite half… and also you get a little bit of powdery residue so you’re a bit, you want to get everything you can really because it's such a small dose you want to make sure she is getting as much as she needs.” AiF-P025 [INACCURACY - POWDER] • “Urmm … you get a half from it, but you don't get exactly 50%. You’ve just got to try your best and hope it doesn't crumble.” AiF-P005 [INACCURACY] • “I don't know whether it's the drug because it's just to prevent a urine infection it's not actually; it's not a hugely important tablet. It is important that she gets the dose, but I don't know, a few milligrams either side in my head, I don't think ‘oh gosh this is doing her serious damage’.” AiF-P006 [SIGNIFICANCE OF MANIPULATION INACCURACY] • “It's either a little bit under or little bit over or one crumbles so then he goes a day without having it or then I come to the end of the month, and I fall short.” AiF-P009 [MISSED DOSES/SHORTAGE] • “We found a lot of it stayed in the thing [tablet grinder] and it was hard and messy at times, trying to get it in the pot.” AiF-P034 [HASSLE] • “Sometimes it would crumble up or something, so you weren't exactly giving them half, so I found it easier just to dissolve it [Proportion of liquid now taken for required dose, instead of cutting the tablet in half].” AiF-P003 [PERSONAL APPROACH] • “I just break it by my hands. We used to use tablet cutters before, but the thing is with the tablet cutters what they used to do is erm, I used to find that when I’m trying to break it in half, three quarters of the tablet would have been broken but the other quarter would have been just left, into like powdery form. So, I just found it better just breaking it with my hands.” AiF-P027 [PERSONAL APPROACH] • “We got a cutter to cut them in half, which never ever cuts them in half, it always goes in bits. So were wasting more than we’re using really.” AiF-P007 [EQUIP/WASTE] • “When they give me that [pill cutter], I thought it would be easier but it's not, no. It's just frustrating. That's what it is.” AiF-P009 [EQUIPMENT] • “So, it wasn't like it's got to be as exact, like Aspirin. And so, we just tend to break it and give him about half.” AiF-P005 [VITAL MEDICINE] • “So, it's not ideal and it is a bit of a hassle but from what we’ve gone through; it's nothing.” AiF-P006 [ACCEPTED IT] • “They’ve always had that line on, and I’ve always tried to cut it on that line, and I just thought ‘oh maybe that showed you where the half is’, but I’ve never really thought about it.” AiF-P033 [SCORELINE]

**Table 5:** Theme: unlicensed medicine and off-label prescribing additional participant quotations.

Key theme	Additional quotations
Unlicensed medicine and off-label prescribing	• “Liquid Omeprazole was a nightmare. I used to have to order it about two weeks before because it was a liquid, and it just took ages.” AiF-P034 [UL MED - DIFFICULT TO OBTAIN] • “Well, I’ve had quite a lot of trouble trying to get most of these drugs from my GP.” AiF-P034 [UL MED - DIFFICULT TO OBTAIN] • “Having to come and pick it up from the hospital and things like that you know with a young family is not ideal.” AiF-P005 [UL MED - DIFFICULT TO OBTAIN] • “They were just hard to get hold of and so I had to order like two at once because they would take a couple of weeks for them to come but for whatever reason they would only prescribe him 14-days’ worth, so I was going back to the chemist every couple of days.” AiF-P033 [UL MED - DIFFICULT TO OBTAIN] • “So normally we would have to go to the hospital once every month or something to pick it up and we don't drive so it's quite awkward. So, my partner was asking if she could have three months’ worth of meds each time we go, so were not having to make the trip as much. So obviously the easiest way of giving the Fludrocortisone is a tablet form which means obviously, you know, we can get 3 months’ worth of her meds at a time. […] My family all live in Doncaster, so it's only [Partners Name]'s family that we can rely on and when we can't you know it's a bus, a train, another bus, another train, and another bus. It takes like 8 h you know just to get there and back.” AiF-P016 [UL MED - DIFFICULT TO OBTAIN] • “You can't just get it because it's a special.” AiF-P005 [UL MED - DIFFICULT TO OBTAIN] • “I know at one point there was a mix up with her drugs. I’d ordered prednisolone, the dispersible tablet from the GP, but the GP on the prescription to the pharmacist wrote 6 × 5 mL, not 5 mg tablets. So, then the pharmacist got liquid prednisolone made up, which took forever […] it cost an absolute fortune.” AiF-P034 [UL MED - DIFFICULT TO OBTAIN/PRESCRIBING ERROR/HIGH COST] • “Obviously, if it was designed for children or for young people, it would put your mind at rest rather than being told were going to put them on this medicine, but it's not designed for them so were going to keep an extra eye on them because then that brings up all kinds of anxiety issues, like about what could happen, what could go wrong and things.” AiF-P026 [UL MED - STRESS/ANXIETY]

**Table 6:** Theme: framework of appropriateness additional participant quotations.

Key theme	Additional quotations
Framework of appropriateness	• “Erm I mean if it was a liquid it would still be once a day, but we would know he was getting the right dose whereas I have to constantly think ‘oh I’ve got to split the tablet’.” AiF-P033 [AaF DESIGN - WANTS] • “Even being able to administer like a quarter of a tablet that has actually been manufactured in a quarter or in a half so when they do go up, they are already [available] in that dosage, so that you know that you’re giving the right dosage rather than it crumbling or splitting.” AiF-P033 [AaF DESIGN - WANTS] • “Well when he is 10 kg he will go onto a full tablet, which will be nice and easy.”AiF-P033 [AaF DESIGN - WANTS] • “Yeah, so a 50 and a 25 […] I think you need that smaller one just to break it down a bit, yeah.” AiF-P007 [AaF DESIGN - WANTS] • “It would be good if you could condense them down, especially when the kids’ having a lot.” AiF-P003 [AaF DESIGN - WANTS] • “20mls is a lot of liquid to give him. So, I guess volume wise as well you need to think about that.” AiF-P005 [AaF DESIGN - WANTS] • “I think a liquid because it is just like drinking juice and it's just like her drinking a drink. Liquid form would be easier but something that tastes not like a medicine, something that tastes nice. I know some antibiotics are banana flavoured or strawberry so you know something like with an additive, like a flavour.” AiF-P032 [AaF DESIGN - WANTS] • “I think they are more suitable because they come with a syringe, they are in liquid form, you know exactly what you’re giving them and for instance, if they don't take the full amount, you know exactly how much they’ve had.” AiF-P033 [AaF DESIGN - WANTS] • “Maybe she needs something more paediatric, something nice. I know it sounds silly but something maybe… well maybe something with stickers on or something to make them want to use it [Talking about inhaler/spacer device].” AiF-P032 [AaF DESIGN - WANTS] • “Yeah, a lot of them are ‘cos you can get the liquid form, the tablet form, the soluble form and their usually pretty measured out because if you can't get the right dose in a tablet, you can get liquid.” AiF-P007 [AaF DESIGN - WANTS] • “Its money isn't it and it's got to appeal because you want someone to buy it. You know if there is one thing on the shelf then they’re going to buy that but if there's two things and one pitches nicely at a kid with a teddy bear on then it's gonna appeal isn't it.” AiF-P021 [AaF DESIGN - WANTS] • “Obviously, the tablets that you don't need to do anything with, like for hay fever and Nurofen capsules and things like that, were you’re just handing him a capsule, it's actually a lot easier but chopping a tablet, it's not a major issue.” AiF-P026 [AaF DESIGN - WANTS]

**Table 7:** Theme: knowledge, training, communication, and relationships prescribing additional participant quotations.

Key theme	Additional quotations
Knowledge, training, communication, and relationships	• “I had a brief sort of outline about what it was being prescribed for. It made perfect sense to me as to why that was being prescribed because obviously you don't want the heart to be enlarged by excessive pressure.” AiF-P021 [GATHERING INFORMATION/KNOWLEDGE] • “Well, I like to know exactly what is being prescribed.” AiF-P033 [GATHERING INFORMATION/KNOWLEDGE] • “I don't think we were shown, maybe we were? This is the thing were…. you’re talking about a very traumatic time, so we might have been, and it might have just gone out of my head. I’m quite aware of that but obviously; if we were it wasn't enough.” AiF-P005 [LACK OF TRAINING] • “You get an instruction over-the-counter but it is quite different in practice.” AiF-P005 [MORE INFORMATION/TRAINING NEEDED] • “They always decided to tell us things later though, like when she was first born with the sodium, they never mentioned about putting it into her milk or anything, they just said she needed it [..] And then they said you can just pop that in the milk and we were like ‘oh thanks’.” AiF-P016 [BETTER COMMUNICATION - MORE INFORMATION NEEDED] • “Erm but it's not a massively essential drug, so I think it's a statin […] I did mention it to the doctors, but they said oh you know, don't worry too much about it.” AiF-P034 [REASSURANCE BY PRESCRIBER] • “I suppose it was from intensive care she's been on a lot of these, and they were just automatically, I suppose because she had an NG tube they were given as liquid and I suppose, because she's a child, and children don't generally take tablets and because I don't think she would take a tablet either, that's why they have all been liquid.” AiF-P034 [POOR COMMUNICATION – NO INCLUSION OF FAMILY IN PRESCRIBING DECISISON] • “Well, we’ve been told before off the GP, because of her age, when she turned two that the liquid form would be taken off her and she would be put on the tablet form.” AiF-P032 [POOR COMMUNICATION - NO INCLUSION OF FAMILY IN PRESCRIBING DECISISON] • “When she first started being prescribed medicines in tablet form, we did struggle a lot and we did tell them, but they wouldn't give us any other form or any other way of getting it into her.” AiF-P032 [POOR COMMUNICATION - NO INCLUSION OF FAMILY IN PRESCRIBING DECISISON]

**Table 8:** Theme: patient and family experience additional participant quotations.

Key theme	Additional quotations
Patient and family experience	• “With what she takes, she's fine; she's a real star actually.” AiF-P006 [GOOD COMPLIANCE] • “Fab she is, yeah fab […] she knows it's her heart medicine and it's so it doesn't stop going ‘bum bum’.” AiF-P021 [GOOD COMPLIANCE] • “Because of her ASD pathway and she's got sensory issues, so we did struggle trying to get a child with all her issues to put something in her mouth and do that.” AiF-P032 [DAILY LIFE CHALLENGE] • “So yeah, we’d just make sure he got his Aspirin first that was the main one and then after that we would obviously; give him the nicer one [last].” AiF-P027 [PERSONALISED STRATEGY] • “I think with experience and just figuring out what works best for him and what makes the medicine dissolve a lot better. I just realise that if I do it in warm water, it's gonna dissolves properly and I’m not really going to have issues with it.” AiF-P027 [PERSONALISED STRATEGY] • “Yeah, so I make sure there in a pot for her as well because I don't know if she’d be able to because she's got… well her fingers are like twiglets, their absolutely bizarre, so I don't know how she would cope with it… So, it does make me feel I need to be prepared.” AiF-P006 [PERSONALISED STRATEGY] • “Both my girls before they go to bed have chocolate milk, hot chocolate and so I suppose it was me, I just stuck it in there just to get it down her. She has had it from the syringe in her mouth before, but she’ll say yuck. So, it just gets stuck in the chocolate milk.” AiF-P021 [COVERT ADMINISTRATION] • “With the Omeprazole it was 10 mL, so she used to call it the big one, because it was the biggest one and it was unfortunate, it was also the foul tasting one as well.” AiF-P034 [TASTE ISSUES] • “The calcium carb is the one to remember that you’ve got to take out with you for her feeds, so you’ve got to carry medicines out with you.” AiF-P003 [DIFFICULTY TRANSPORTING MEDICINES] • “When I’m at home it doesn't really make much difference it's just when we travel that it's a bit of a nuisance.” AiF-P027 [DIFFICULTY TRANSPORTING MEDICINES] • “I do think going away is more difficult […] for me I worry that I will drop the Tacrolimus bottle and then we’ll be in Spain, and we won't have any.” AiF-P034 [DIFFICULTY TRANSPORTING MEDICINES] • “No, we just had to sign a form through the SENCO [special educational needs coordinator]. They are quite good with [Patient Name], with her background, their willing and they will do anything for her.” AiF-P032 [MEDICINES IN SCHOOLS] • “So yeah, we decided against getting the nursery involved, which is why we do it in the evening.” AiF-P025 [MEDICINES IN SCHOOLS] • “Yes, especially if it was like a heart med, you’d want to know, as you can't miss that dose and you need to know that everything has gone in to help them.” AiF-P032 [ANXIETY] • “It is a worry knowing if they are having enough, ‘cos she does still have the SVT attacks.” AiF-P007 [ANXIETY]

### 3.1 Theme 1: inappropriate formulations

Participants observed that the medicine provided for their child was “designed for adults” as opposed to children. Furthermore, they reported that the pharmaceutical design of the dosage form was unsuitable for their child's needs as they were either having to “cut” or “dissolve” tablets and/or “open” capsules to obtain the required dose, which caused difficulties in handling, longer preparation times, and frustration.

“It's more a pain because I’ve got to take my time; ‘cos I’ve got to like really concentrate and do it quite slowly.” AiF-P009

The main concern was the accuracy of the dose following manipulation owing to the tablet not being cut “exactly” or it “crumbles to bits”. Powder losses on transfer during administration were frequently noted.

“She doesn't get 100% of the dose, because probably some of it stays a bit on the spoons and some of it maybe spills out when I’m trying to get it in the cup and some of it … so you know, in terms of being an accurate dose, it's not very accurate.” AiF-P034

This imprecision resulted in repeated preparation of doses, mess, medication wastage and a feeling of uncertainty, if the manipulation did not go as planned.

“If I’m not satisfied on how I’ve done it or if it's missed the mark, I tend to bin the whole tablet and start again.” AiF-P006

Participants had an awareness about the importance of accuracy and the significance of this related to the medicine's indication. They related their level of concern to the type of drug prescribed, prioritising some medicines as more vital than others.

“Erm but it's not a massively essential drug […] I did mention it to the doctors, but they said, ‘oh you know, don't worry too much about it’.” AiF-P034

“It's not a hugely important tablet. It is important that she gets the dose, but I don't know, a few milligrams either side in my head, I don't think ‘oh gosh this is doing her serious damage’.” AiF-P006

The complexity level of the manipulation technique performed by participants was also raised as an issue from previous experience, as in the past they had found it more challenging to quarter tablets rather than halving them. However, participants did feel more confident with time and practice.

“The quarter was really, really difficult.” AiF-P027

“It was stressful when he was on a quarter but now, he's on half, it is a lot easier and when he goes onto a full tablet it will be even easier still.” AiF-P033

Participants described using personalised approaches to adapt their method of preparation and administration techniques to find a solution that worked “best for them” as a family, and felt it was “more accurate” this way. As a result, parents found their own alternate ways such as breaking tablets by hand or using a kitchen knife to cut them instead. The lack of standardisation in terms of equipment across different hospitals and within the community had also caused administration device compatibility issues.

“We’ve managed to find our own path of how we do it. It's a faff, it's a pain really but it's part of our life and its necessary and so we just have to get on with it.” AiF-P005

Some participants recognised that the manipulation required had to be done as there was no alternative available and so had accepted it, even though they could see it was not “ideal”.

“You’ve just got to work with what you’ve been given to give them.” AiF-P016

Participants also recalled trying to use the remaining parts of the dosage form, retaining them for their child's next dose, where possible.

“Yeah, I use the remaining half for the following day.” AiF-P027

Manipulation of the available medicine was described a source of anxiety by some families due to the variable dose being received and the potential risk of under/overdosing. This raised concerns about the possible consequences of this such as treatment failure or increased chance of experiencing dose-related side effects. Also, having to seek further supplies from the prescriber when manipulations “went wrong” was highlighted as another stress factor.

“And it was always really frustrating because you never felt like she was getting enough.” AiF-P025

When seeking further medication supplies, medicines obtained were often noted to be from different manufacturers and this regularly led to noticeable changes in the design of medicine supplied (such as flavour, colour, size, shape or if it was scored). The purpose of the score-mark on the tablet was unclear to most of the participants, whilst for others its presence was noted as making a big difference to the “success” of the manipulation performed.

“You know the ones that didn't have a line down the middle; I noticed those tend to crumble. I guess it's because of the force when you cut it in half.” AiF-P027

When appropriate formulations are not available, there is an increased risk of medication errors occurring, both in terms of prescribing and dosing.

“We ended up with the tablets because we were running out of it […] I thought well we’ll do the same as what we do with the pravastatin and crush it. But obviously, the dose was different, and I just went on automatic pilot and crushed the whole tablet, like I do with pravastatin, gave it her all and then realised the dose was higher than I should have given her, and I had this massive panic.” AiF-P034

Participants reported having more confidence in the efficacy of AaFs in comparison to AiFs. However, it was recognised that having an AaF was associated with increased prescribing costs.

“It's cheaper in a tablet form than what it is to make a liquid form or prescribe a liquid form.” AiF-P032

### 3.2 Theme 2: unlicensed medicine and off-label prescribing

Any authorised (licensed) medicine being used in a different way to that which is described within its MA is said to be used “off-label”. An example of this is when a medicine is used to treat a patient who is in an age group outside its licensed age range. An UL medicine is one which does not have a valid approval in the country in which is it prescribed/administered (30).

Bound by ethical restrictions, this theme could not be raised as a topic for discussion with participants during the interview unless they had prior knowledge of this from previous discussions with their HCPs, to avoid any alarm or distress to the families involved.

Consequently, only three of the 13 parents (23.1%) interviewed appeared to be aware that the medicine being taken by their child was an ULOL medicine and raised some concerns regarding this. However, all the children in this study were receiving either an UL or OL medicine (or both in some cases). This is because when licensed medicines are manipulated or modified in an unauthorised manner, they are being used OL. Most of the parents had obviously not considered this before and were completely unaware of their medicines being used in this way; having simply followed the clinician's dosing instructions.

“It was just hard to get hold of them just because they were on the specials list […] You can't just get it because it's a special.” AiF-P005

The use of UL medicines (or OL use) appeared to be a cause of added anxiety for families, as parents wanted reassurance that the medicine provided was safe for their child to take.

“I don't know whether this is right, this is what a pharmacist said to me; it's not licensed in children under 16 […] so we had a lot of reservations about her going on Aspirin.” AiF-P025

One participant on reading the patient information leaflet (PIL) supplied with their child's medicine had noted that it should be 'swallowed whole', and this was different to how her child was taking it.

“Well with the Melatonin, the GP won't prescribe it […] because the GP refuses point blank to prescribe it […] And she is on 4 mg slow release, but we find it hard with them because when you read the package it says they can't be [chewed] … Well, they have got to be swallowed whole.” AiF-P032

Parents reported finding it difficult to obtain UL medicines, from their GP as they were unable or unwilling to prescribe medicines that had been commenced by a specialist paediatric hospital.

“Well, I’ve had quite a lot of trouble trying to get most of these drugs from my GP.” AiF-P034

A lack of communication was reported between the GP and family in terms of who was responsible for prescribing each medicine (particularly UL medicines) often leading to prescription requests being rejected, without the parent being notified, causing prescription delays.

“But sometimes what would happen is the GP would say with the Azathioprine, ‘I’m not licensed to prescribe that’ so would send it to the chemist, without the Azathioprine.” AiF-P034

Parents described difficulties in obtaining UL medicines either directly from the hospital (which may not have been local to them) or needing to order it in advance via a community pharmacy from a specials manufacturer, which took additional time as these medicines are not held in stock.

“I had a fight on with the doctors because they were like ‘well you should have enough’. And I was like well they don't order it in time, as it takes them a week to get it in and get it ordered. So, it was quite a battle but because he needed it, there was nothing we could do, so they did accommodate but it was tough.” AiF-P033

This caused “hassle” and inconvenience for the families having to constantly forward plan the re-ordering of these medicines due to the ‘anticipated delay” in supply; resulting in parents having to make special journeys to obtain further supplies of their child's repeat medication.

Yeah, it's a pain […] I think of my mental health and try to spend as much time away from it [the hospital] as much as I can [laughs]. But we do have to come back here once a week to get the medicines.” AiF-P005

There was also a general “fear factor” surrounding the use of liquid specials, regarding dropping or running out of them. One parent found it a constant worry, aware of the fact that she would find it extremely difficult to obtain further supplies.

“These medicines are keeping her alive basically so I can't run out of them, you know. I have a real panic if I drop the Tacrolimus bottle, I think ‘oh my god’ […] and if we go away, I only kind of take what we need.” AiF-P034

One parent also reported that they had chosen as a family to manipulate the tablet formulation of fludrocortisone due to the inconvenience of having to collect their monthly supply of the “child-friendly” liquid formulation (which required extemporaneously preparation by AH Pharmacy) as this product had a limited expiry date and needed to be stored in the fridge. The distance that needed to be travelled to obtain the liquid form was the main decision factor for them as they often felt dependent on other family members because they could not drive.

“So normally we would have to go to the hospital once every month or something to pick it up and we don't drive so it's quite awkward.” AiF-P016

Differences were noted by parents and children when different “batches” were supplied for liquid 'specials', due to variation in the formulation manufactured, depending on the source of the UL medicine.

“I don't know whether they have two ways of making it up, but some batches […] there was one that was particularly more yellowy than the other one and she hated that.” AiF-P034

### 3.3 Theme 3: framework of appropriateness

Firstly, parents reported wanting paediatric medicines that were easy to use, having a simple administration process. The “ideal” formulation needed to be ready-made, in an appropriate strength for a child, which contained a suitable dosing device (where applicable) to ensure accuracy of the dose given. It was extremely important for the medicine to have a taste nice, be flavored and not smell horrid. Small dose volumes were also desirable.

“The likes of that are easily administered, easily accessible and easy to prepare.” AiF-P005

“Something that would disperse in the mouth, something that tastes nice, something that wouldn't make a child sick or gag and making sure that the full dose was going in.” AiF-P032

Widespread availability was vital. Readily available paediatric medicines were sought by parents to lessen the need for reliance on supplies only available from specialised hospitals or other ’special' manufacturers due to the inconvenience associated with trying to obtain these.

“If it was a lot more easily available because at the minute you can only get it at one hospital, Alder Hey.” AiF-P016

To resolve the inaccuracy issue, they sought medicines that did not have to be manipulated at point of administration so they could be given easily, in the way it comes. Most of the parents agreed it would be preferable for an alternate formulation to be available (rather than the one they were currently administering to their child), so they did not need to manipulate it before administration. They wanted a medicine of an appropriate strength for a child, which provided an accurate dose, so they could assure the correct amount needed was received each time.

“If we didn't have to cut that it would be ideal.” AiF-P006

“So, if it was made in the right quantity [strength] already then you wouldn't have to do all that.” AiF-P026

Liquid medicines were deemed to be the easiest to give, especially to younger children by most of the parents interviewed and so parents wanted liquid formulations to be available, or at least a dispersible option.

“If someone said right you can have either have liquid or tablets, they work exactly the same, what would you go for? … I would go for liquid, purely because when you draw it up you know exactly how much is in there.” AiF-P033

In comparison, other parents thought tablets were much more convenient than liquids.

“Erm since he's been on tablets I think it's so much easier. Even just standing there, pulling them up and there is a danger if you dropped the liquids, but if you drop a box of tablets, you can pick them back up again… but with liquid it's gone and it's going the doctors again.” AiF-P009

One parent had switched away from liquids for her child now that she was a bit older to help make her feel “grown up” and avoid any embarrassment in front of her peers.

“We are trying to make her feel as grown up as possible and make her look as grown up as possible, so we thought the tablets might be a really good idea.” AiF-P006

The size of the solid oral dosages forms (SODFs) such as tablets and capsules was also a concern and needed to be considered as parents acknowledged the difficulty particularly for younger children when having to try swallow large sized dosage forms (DFs).

“The size of certain tablets what kids have got to take as well. I definitely think either smaller for certain ages […] something melting that can melt or disperse in the mouth and something that tastes nice and it doesn't taste like you’re taking a medicine.” AiF-P032

Having medicines (and medical devices) which were attractive and appealing for children to use was discussed. Some of the parents acknowledged that medicines purchased over-the-counter (OTC) in pharmacies or supermarkets had been purposefully designed for children to use and so they were much easier to give. They liked the fact that it was the “right dose” and could be measured out conveniently. The wider choice of DF options available OTC to suit children of different age groups was also noted. One parent appreciated this was all about cost and that the item on the shelf needed “to appeal” to the consumer so they would purchase it.

“The things you normally get over-the-counter, things like Benadryl allergy stuff and Calpol are so much better because they are like you say designed for a child. The doses are clear what you need to give them. They normally come with something to administer them with i.e., syringes and they’re flavoured and even like the texture of them, they’re more like a bit gloopy.” AiF-P025

### 3.4 Theme 4: knowledge, training, communication, and relationships

All parents interviewed showed a good level of understanding about their child's current diagnosis and the reason why each medicine had been prescribed for their child.

“Yeah, I think we’ve always been provided with information, and I think we’ve always asked a lot of questions. I’m in Healthcare as well, in my profession, so you know it's something I would have always done.” AiF-P005

However, it was clear that the level of medication advice, training and support provided by HCPs had varied across paediatric hospital sites/wards prior to discharge home.

“When we were leaving hospital, the nurse did say it's just half a tablet, break it in half and put it in water and give it to her and that was about as far as it went so it was a bit of a casual chat.” AiF-P025

This ranged from some receiving very clear, structured practical demonstrations of how to prepare each medicine including important medication safety checks (such as double dose checking and reviewing the expiry dates) to others simply being left to figure it out for themselves, or finding advice was only offered by HCPs after an administration issue had occurred.

“They give you sort of training as parents about the drugs, what they do and how to administer them … part of what they train you is always check the dose, always check the sell-by date.” AiF-P034

“No, they didn't show us. They just handed it to us and then when we came home, it was kind of figuring it out for ourselves kind of a deal. But yeah, we just had to figure it out for ourselves.” AiF-P027

Parents spoke of searching for answers online themselves or later sharing their own personal experiences with others in a similar situation to offer support and share their knowledge, once they had figured out the best way for them.

“And actually, looking back now, as a healthcare professional and as a mum that's been through it all, you want to support other mums who are going through the same thing and advising them on… saying ‘try this because that helps’… and actually ‘no, you shouldn't know how to do it all’. So actually, I think there's a gap there.” AiF-P005

Written information and verbal advice were valued. Some parents found the lack of communication particularly infuriating and wanted simple, practical advice to be offered as standard to every family prior to starting any new medicines.

“Well, if it was fine why didn't you tell us that, you’re telling us now and that was like a year after we were given it when they mentioned it; so, we were making sure we had an alarm set to do it and get up at 12 [Midnight] if we went to bed before.” AiF-P016

Knowledge did appear to empower the parents and helped them to make informed decisions about their child's care. Parents were reassured by the provision of medicine administration training by staff and felt more confident with regards to dose preparation at home when this form of education had been offered.

“Yeah, the staff nurse on the ward he was on, he was on the cardiac ward, and they basically said this is how we would do his tablet […] so it was nice to know, that I knew what I was doing at home.” AiF-P033

Parents also listened to any guidance offered by HCPs to overcome administration issues experienced such as switching to an alternate dosage form type such as tablets/capsules, trying co-administration with certain foods and drinks to ease administration and/or mask palatability issues or using an alternate route of administration (RoA) for problematic medicines such as those given via enteral feed tubes.

“It just doesn't dissolve at all and initially [Named Specialist Nurse] suggested like crushing it, but it's got like a plastic coating on the outside or sticky coating, so it doesn't crush well […] so I don't crush it now as crushing it doesn't work. And so, the only way I have found to dissolve it; is in warm water.” AiF-P005

“For the nasty ones we used to put them down her gastrostomy.” AiF-P006

Parents trusted that their HCP had provided the most appropriate and suitable DF available for their child. A lack of shared decision making was reported. They were not included in any form of discussion to advise on appropriate formulation options available prior to their child commencing the new medicine(s) prescribed.

“I had no conversation with the doctors. The doctor told me that she was going to give it to me in a liquid form just purely because it would be easier to get it down her neck. Obviously, she was only two when she started with it so there was no way she was going to swallow a tablet at that age.” AiF-P021

In terms of medicine manipulation, there appeared to be no clear guidance given. Parents were using different methods and techniques to complete the manipulation, with some for example taking a proportional dose, whilst others cut the tablet first.

“I mean looking back, it wasn't all going into her mouth, and it was quite tricky at the beginning to try and figure out the best method. And there wasn't a manuscript, and nobody was telling us how they were doing it, so we were sort of figuring it out for ourselves.” AiF-P025

One parent had even set up her own “pill school” training sessions at home to help her child learn how to swallow a tablet, which had been encouraged by the clinical team.

“Before her transplant, we started, because of those massive MMF ones; we were practicing with tic-tacs for her, so started with half a tic-tac to get her to actually take it. Because up until that point, I don't think she was on many tablets, it was mainly liquids, so we were just trying to make her life as easy as possible for her.” AiF-P006

### 3.5 Theme 5: patient and family experience

At home, parents are often responsible for the daily management and administration of medicines to their child as children begin to take responsibility for their medications at different ages, and this can vary greatly. Most parents reported that their child currently demonstrated good compliance, showing a general willingness to take their medicine(s) without any issue. They recognised the importance of taking these medicines to keep them well but did acknowledge needing to perform a medicine manipulation in the future may also be challenging for their child, and they hoped there would be more suitable formulations available by then.

“She's incredible at taking what is quite a disgusting medicine as it is horrid, and it doesn't taste nice at all. It's horrible like… very powdery but she is very good at taking it.” AiF-P025

“Well, I think it's hard work because I have to keep my eye on her because I never know … well she doesn't get the half and the quarter thing and so you’ve got to watch over it, when she's old enough really to just be doing it and taking her own. And she does take her own, but I have to watch with the half thing.” AiF-P007

All parents spoke of the daily challenges faced when managing their child's medicines at home, recalling past issues which had caused a real impact on family life at the time. Nonetheless, parents expressed that giving medicines to their child had now just become part of their “daily routine”.

“Yeah, it's become everyday life, so for the last year we’ve done it.” AiF-P033

Giving medication to a child, particularly a young baby, requires acquisition of skills and confidence. Many parents reported feeling unsure to begin with, but over time learnt from personal experience and from others. This offered them reassurance going forward, particularly when new medicines were started.

“I didn't want to give him a lower dose or a higher dose, so I was a bit unsure how much I was giving him.” AiF-P033

“My mum had her one day, she used to be a nurse years ago and she just used a spoon, and I thought well actually that's a lot better than what I’d been doing, so that's why I do the spoons.” AiF-P034

One parent spoke of her difficulties with so called “authorised” manipulations as even these approved methods had caused difficulties on administration for her family in the past, finding them inconvenient to use, and suboptimal.

“We had to open the capsule and pour it on his food and if you were out and about and he wanted a snack it was a nightmare, it was like being back with the scoop, it would just go everywhere. And then obviously sometimes he would have a couple of bowel problems because he wasn't getting the full dosage as well because it was blowing away or whatever.” AiF-P009

Personalised strategies had been developed by individual parents to overcome previous medicine administration issues. The ordering of medicines dependent on their taste was a common technique used by parents to persuade their child to take their medication with the nicest one (most palatable/favourable) being offered last to wash it down.

“Did I tell you about the Amlodipine, she really doesn't like that. She sort of screws her face up but I always try and give her… well we finish with the Aciclovir, so I give her the one she likes after that.” AiF-P034

Other strategies identified included the development of their “own” improved preparation and administration system.

“Yes, I feel a lot more confident now we’ve got the silicone cup because when we were doing it with the metal spoon or with the syringes, I wasn't always confident she was getting the right dose.” AiF-P025

Pre-preparing doses needed in advance was another way parents used to stay organised, increase safety by reducing the risk of distractions, and decreasing the total time taken preparing medicines each day, especially whilst in a busy home environment.

“In the end, I bought the freezer bags from IKEA because they were quite sturdy and could be labelled, with the times on for the day so I made them all up for the day and just pulled them out, so I developed that system.” AiF-P005

Other ways reported included co-administration with foodstuffs for ease and using an alternate RoA to avoid any hassle.

“We’ve got that option obviously giving it down his gastrostomy.” AiF-P027

Some described using covert administration techniques mixing it into a drink before bed, with the child being completely unaware they were taking a medicine.

“We have to sneak it. She doesn't realise that it is medicine; she doesn't watch us put it in. Then we tell her you’ve got to drink your drink now before you go to bed.” AiF-P032

Some of the parents realised that their child had specific issues relating to their own personal circumstances. For example, some were physically unable to swallow tablets or capsules due to their current diagnosis.

“Well part of her condition with Williams is she's got a hypersensitive mouth, so, she's very sensitive to textures and she’ll only eat certain foods. So, she wouldn't take tablets. Erm, so all her medicines are liquid, or dissolved, or crushed and mixed with water.” AiF-P034

Ranitidine was reported as a medicine which had poor acceptance due to its taste by some of the parents, but other medicines also caused issues due to their taste such as omeprazole. An alternate medicine needed to be prescribed.

“She couldn't take the ranitidine with the taste, there was a fight trying to get it down her, so we changed it.” AiF-P032

The struggle of having to navigate the healthcare system when trying to obtain further supplies of their child's medication was particularly demanding for many parents, leaving them feeling overwhelmed.

“Obviously if it breaks then I worry like ‘oh I’ve got to go and ask the doctors to see if I can get them early’. And trying to get it out of them is a nightmare.” AiF-P009

The logistics of travelling outside of the home with medicines had the most significant day-to-day impact on families, especially if medicines required specialist storage conditions or were bulky to transport. Holiday travel (especially if going abroad) also caused added concern for families regarding transporting medicines and seeking further supplies, if things went wrong.

“I think as a mum it's how you go out and about in the day with the medicine. You don't want to take the bottles out with you because it's a big glass bottle.” AiF-P005

“I do think going away is more difficult […] for me I worry that I will drop the Tacrolimus bottle and then we’ll be in Spain, and we won't have any.” AiF-P034

Generally, parents with school-aged children shared having a positive experience with their child's school if medicines needed to be administered during school hours, providing the relevant documentation had been completed.

“Most schools don't like to give them; you’ve got to go up and give it him yourself. But they are quite good like that though, so they have just added it into his care plan.” AiF-P009

However, others described needing to alter the time of administration to work around the school day as avoiding the need to involve the school altogether was the easiest way to get around this issue.

“I think if you expect the school to give them a drug, then you have to go in and fill out a form and it has to be stored in a place and I just thought [frustrated sigh] […] just leave it until after school, that's fine.” AiF-P034

For younger children attending nursery, parents likewise avoided the need for medicines to be administered in their absence by adjusting administration times once again as they wanted to be solely responsible due to the complexity involved.

“Because of all the equipment and the process, I probably wouldn't be happy with anyone else outside of the family doing it to be honest, just because it is a pain. And because the dose is so small, you think if any of its lost; it's actually dosage wise, quite a big bit if you know what I mean.” AiF-P025

## 4 Discussion

To our knowledge, this is the first study to provide an in-depth qualitative exploration of the challenges faced by parents and carers when administering AiFs to children at home. The findings reveal that AiFs present substantial practical and emotional burdens for families, which are further compounded by the everyday demands of caregiving. Importantly, these experiences varied according to the child's developmental stage, with different challenges reported by parents of younger children compared with older children and adolescents.

**Figure 2:**
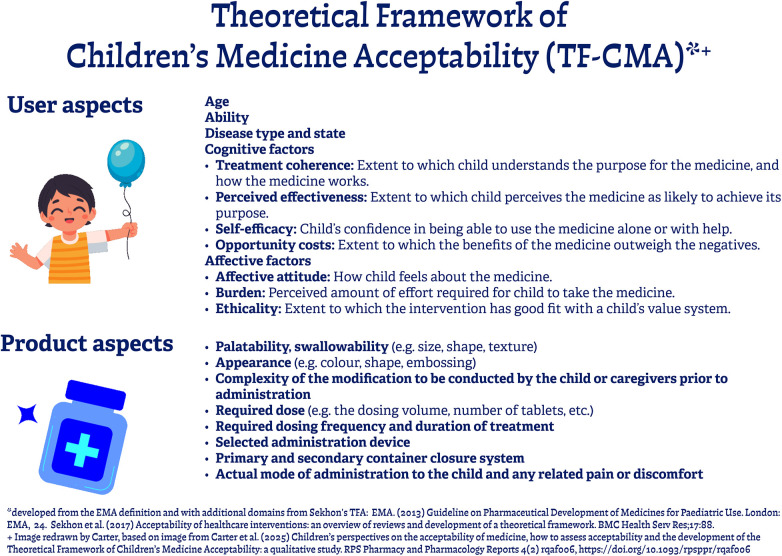
Theoretical framework of children’s medicine acceptability (TF-CMA).

Although this study was not designed within a theoretical or conceptual framework, the findings resonate with several dimensions of a recently proposed framework, the “Theoretical Framework of Children's Medicine Acceptability (TF-CMA)” (31). The TF-CMA builds upon the constructs and components proposed in the Theoretical Framework of Acceptability (32, 33) and the European Medicine Agency guideline (34). The TF-CMA framework was developed to support insight into children's perspectives on medicine acceptability but it also offers interesting opportunities for considering parents' perspectives on administering medicines to their children. The TF-CMA (Figure 2) comprises two overarching domains: “user aspects” and “product aspects”. Broadly “user aspects” include age, ability, disease type/state, four factors relating to cognitive factors (treatment coherence, perceived effectiveness, self-efficacy, and opportunity costs) and three relating to affective responses (affective attitude, burden, and ethicality). The “product aspects” domain includes factors such as palatability, appearance, complexity of modification, dosing regimen, devices, containers and mode of administration. These domains provide a useful lens through which to interpret the findings of this study.

Parents reported that administering AiFs was significantly more time-consuming and demanding than administering AaFs; this links to “burden” in the TF-CMA, where the level of effort impacts or a parent's feelings toward the medicine. Dosing often required extensive preparation, precise manipulation and considerable forward planning, which led to concerns about accuracy and increased risk of error. There was significant variation in the complexity of measures taken to manage AiFs, differences between families were highlighted—some expressed significant anxiety while others appeared to be reconciled to the required actions; this links to “affective attitude” within the TF-CMA, which acknowledges that experience (both negative and positive) can shape feeling towards medication. Many families report difficulties obtaining AiFs from primary care, or even hospital. Successful use of AiFs depended on strong habits which could lead to errors if one habit was applied to another medicine. With practice, families developed ways to cope with the need to administer AiFs; linking with 'self-efficacy' within the TF-CMA. They developed standard procedures that were significant departures from “normal” family life. These adaptations were hard-won but could be disrupted by unexpected changes, for example in the availability of a product.

The families understood the value of thorough communication with HCPs about AiFs and how to manage them, including sharing the importance of each medicine so that families are empowered to make trade-offs when medicine administration does not go to plan; this links to 'self-efficacy' and confidence in administering medication, a cognitive component of the TF-CMA. Difficulty communicating with the primary care team and/or the specialist prescriber was a common problem.

The use of AiFs appeared to be a significant inconvenience for all, with parents agreeing that it would have been preferable to receive a “child-friendly” formulation if one were available; again, another “burden” related TF-CMA factor. It seemed that most of the parents interviewed were accepting, and with time found ways to overcome their initial concerns, typically regarding the imprecision of dosing to minimise any potential associated risks; perhaps, reflecting the “treatment coherence” and “burden” factors within the TF-CMA, where greater understanding of the medication sustains parents' wish to continue with administering it to their child.

Parental perceptions of AiFs seemed to be influenced by their experiences of looking after their child; especially if the drug highlighted was one noted as being vital in “helping to keep them alive”. This assessment of the benefit of the medicine for their child relates to “opportunity costs” within the TF-CMA, where benefits are clearly perceived as outweighing negative aspects. “Ethicality” within the TF-CMA addresses the extent to which the medicine is a good fit with the user's value system, so although parents did not necessarily like their child being prescribed AiF, they accepted that this was the right thing to do. Thankfully, all the children appeared to be doing “well and improving” whilst taking the medicine(s) as prescribed so the parents felt happy for them to continue with their treatment, even though they could visibly see it was suboptimal, with for instance; the subsequent loss of powder when needing to cut a tablet in half or quarters. These experiences relate to “perceived effectiveness” in that they expected the medication would achieve its purpose, despite the challenges.

As highlighted within this study, parents often experienced difficulties administering medicines to children, reflecting on both their current difficulties and previous memorable events; these aspects relate to “burden” and “opportunity costs” as well as to the “product” aspects of the TF-CMA. Taste was the most important factor to achieving successful administration of liquid medicines. Covert administration and alternate strategies were often used to overcome palatability issues. Parents wanted medicines that were readily available, in an easy-to-use format which tasted “nice” and provided accurate dosing on administration. These taste-related findings related to the “palatability/swallowability” and “mode/form of administration” factors within the TF-CMA. Nonetheless, the physical act of modifying medicines to give to children was not a “deal breaker” as families appreciated prescribers often had no choice. Occasionally, the use of AiF was even requested by families themselves on a temporary basis (if they were due to go on holiday) or to help maintain their quality of life (by reducing the number of hospital visits needed to collect medication, particularly 'specials').

In summary, the use of AiFs is “right” in the sense that parents who agreed to be interviewed described how they were able to administer the prescribed medicines to their children. These parents had a pragmatic understanding driven by the imperative to keep their children healthy and therefore developed coping strategies and overcame any initial barriers. However, this does not mean that this situation is optimal, or even desirable. This study was concerned with the parents’ reported perceptions. This study did not intend to investigate the extent of parental anxiety or the nature of other reactions to the situation families found themselves in. Yet, some parents revealed the anxiety/stress caused. Eliciting the burdens arising from AiFs could be difficult because families will want to present themselves as competent. A significant selection bias is possible; families who found it difficult to cope may have been less likely to volunteer for an interview. Nevertheless, this study has shown that considerable burdens exist even if they are incorporated into “business as usual” for affected families. Further work quantifying the burdens faced by families would provide important insight to the value society should place on AaFs and the avoidance of AiFs. Such work could aim to be representative of families and should ascertain burdens in a non-threatening way, perhaps through an anonymous survey that includes other items about family life during chronic illness.

Although the total number of parents included in this study was relatively small, this is typical of qualitative interview studies due to the vast quantity of data recorded and detailed analysis needed thereafter. The recruitment period was also limited. Nonetheless the same themes appeared to continually emerge during the interviews, demonstrating that sufficient data had been collected based on the diminishing emergence of new themes, and the depth and quality of analysis achieved.

An additional limitation of this study was the ethical restriction placed on disclosing the use of unlicensed and off-label (ULOL) medicines unless families were already aware of such use, to minimise this risk of causing distress. This reflects a broader challenge in paediatric prescribing, as parents (and in some cases HCPs) are unaware of the licensing status of the medicine prescribed. There is a clear need for more open and transparent discussions with families about manipulation of medicines and the use of ULOL products, particularly given the limited public awareness on this (35, 36). Clear and proactive communication from HCPs is essential to ensure that parents and carers understand how medicines are commonly prepared and administered in paediatric care. This should include discussion of any implications related to ULOL use, helping families make informed decisions and remain vigilant for potential clinical concerns, such as side effects, particularly when medicines are manipulated to achieve child-appropriate doses. These challenges further underscore the importance of consistent, transparent communication and appropriate formulation choice in paediatric prescribing.

Communication and information-sharing with parents and carers regarding best practices for medicine manipulation remain inconsistent across UK healthcare settings. Findings from this study indicate a lack of knowledge among many HCPs concerning the implications of performing medicine manipulations, as reflected in parental accounts. Raising awareness of existing evidence-based resources, such as the MODRIC guidelines (37), could support HCPs in delivering more standardised, safe, and practical advice to families. Additional interventions, including structured medicine administration training for parents and improved signposting to trustworthy sources of paediatric medicines information (e.g., the Medicines for Children website: www.medicinesforchildren.org.uk), could help build the skills and confidence needed to ensure accurate and safe medicine use at home. Parents in this study highlighted the value of receiving both verbal and written information to reinforce understanding and support effective practice.

Beyond communication, formulation choice itself represents a key modifiable factor in reducing parental burden. The interviews were conducted in 2016/2017 and therefore reflect parental experiences from that period. However, the ongoing lack of progress in the development and widespread availability of child-friendly formulations suggests that many of the challenges identified remain relevant today (38, 39). Since data collection, several advances have occurred, including increased availability of minitablets and dispersible tablets, updated European Medicine Agency (EMA) guidance encouraging the inclusion of alternative administration methods during licensing, and wider dissemination of evidence-based resources such as the MODRIC guidelines (37). Early intervention programmes, such as specialist training clinics (commonly referred to as “Pill School”), have demonstrated success in teaching children—often as young as three years of age—to swallow placebo tablets or capsules using safe training aids such as cake decorations, hard sweets, or dummy pills of differing size.

The prescribing of SODFs, such as tablets and capsules, to children can offer substantial advantages over the equivalent liquid formulations, particularly in terms of cost-effectiveness, ease of storage, and portability (40). SODFs also generally contain fewer excipients, including those flagged as potentially harmful in paediatric populations, and are less likely to present palatability issues. Given the strong association between medicine palatability and treatment adherence in children (41–43), this can have a direct impact on clinical outcomes. Recent advances in formulation technologies, including the development of minitablets, offers promising opportunities to standardise DFs and simplify the experience of families when medicines are used as AiF or AaF. By improving dose flexibility, acceptability, and ease of administration, such innovations may help mitigate some of the challenges caregivers currently face (44).

However, the decision to prescribe a SODF should be informed by a careful risk-benefit assessment, taking into account the need for accurate dosing and the potential pharmacological consequences of DF manipulation in children who are unable to swallow the formulation as intended. It is therefore important that clinicians routinely ask all patients, regardless of age, “Are you able to swallow a pill?”. With appropriate training and support, children may be able to transition safely to SODFs, and clinicians should consider this option rather than defaulting to prescribing liquid formulations (45). Nevertheless, recent reviews of paediatric regulatory frameworks in the US and EU continue to highlight persistent challenges in paediatric drug development, including limited availability of AaFs, inconsistencies in dosing options, and ongoing ethical and clinical barriers (46). Taken together, these findings suggest that, despite technical and regulatory advances, parents are still likely to experience burdens related to inconvenience, time demands, and anxiety when AaFs are unavailable, supply chain issues result in brand changes, or training and support are limited. As such, the insights provided by this study remain important for understanding both the practical and emotional challenges of administering age-inappropriate medicines and for informing future research, regulatory guidance, and the development of child-appropriate formulations. Our expectation of parental knowledge in this area did appear to have been overestimated. Parents trusted their HCP had provided the most suitable formulation available for their child. There also appeared to be a general willingness to follow the instructions given without questioning it unless a clinical issue arose. Most parents had “not really thought about it before” until it was discussed during the interview. This trust in the competence of HCPs to prescribe medicine that would be good for their child is not something specifically addressed within the TF-CMA, although perhaps it aligns to “treatment coherence” and “perceived effectiveness” of both the medication and the professionals.

Several qualitative studies have examined the challenges of paediatric medicine administration from the perspectives of children, parents and carers (47–51), as well as HCPs (52–55). These studies have employed a range of methods including questionnaires, online discussion forums, interviews and focus groups. The findings of our study align with those reported in the existing literature, with the themes identified reflecting common experiences across a wider population. Nonetheless, the primary focus of this study was on challenges associated with the use of AiFs within the home environment. Our findings underscore the emotional and practical challenges faced by parents and carers, and stress the need for clear, transparent communication regarding medicine licensing and the risks of altering formulations. Such communication is essential to support informed decisions and empower parents and carers in managing their child's treatment.

As illustrated in Table 2, the assessment of age-appropriateness for active pharmaceutical ingredients (APIs), such as Trimethoprim and Vitamin K, can vary depending on the specific brand or manufacturer. This variability arises from differences in the methods of administration permitted under the product's MA, as outlined in the Summary of Product Characteristics (SmPC) and/or associated patient information leaflet (PIL). These documents are subject to ongoing updates by the manufacturer whilst the product is being marketed. Parents frequently reported inconsistencies in the brand or manufacturer of medicine(s) dispensed with each prescription, often due to supply chain issues or lack of continuity in sourcing. Such changes were perceived as unsettling, and on occasion impacted the success of the manipulation being performed; within the TF-CMA framework, they align with challenges to “treatment coherence” and “perceived effectiveness”, which can impact “affective attitude”.

By exploring end-user experiences as we have done, including human factor studies of real-world medicine administration, our findings can help the pharmaceutical industry and Health Technology Assessment agencies to understand the burdens and inconvenience caused for caregivers when only AiFs are available. Insights into the alternative administration strategies used by parents (e.g., giving medicines in order of taste by administering the most unpalatable one first) may also be of value to medicine regulators, such as the EMA. Notably, the EMA now encourage manufacturers to investigate and include approved “alternative” methods of administration for their products as part of licensing requirements, particularly for those populations, such as children, who may struggle to take medicines in the conventional way (34, 56).

Emerging evidence highlights the importance of incorporating children's perspectives into medicine acceptability research, as their experiences and preferences can directly inform the design of age-appropriate, child-centred formulations that are practical, more likely to support adherence, and tailored to real-world use (31). The TF-CMA (31) offers a framework—albeit one requiring adaptation from a child- to a parent-focused perspective—that shows promise for prospectively designing research to systematically examine the acceptability of paediatric formulations and administration practices. Building on these insights, future work should quantify the burdens faced by families through representative surveys and continue to explore children's perspectives, as well as prescribers' experiences and decision-making, to guide safer, more effective paediatric care and the development of AaFs.

## 5 Conclusion

This study highlights the burdens families face when administering age-inappropriate medicines, emphasing the importance of increasing access to child-appropriate formulations for essential medicines. Where “legacy” medicines remain the only option, HCPs can support children to safely swallow SODFs, such as tablets and capsules, and educate families on safe medicine manipulation. Further research with parents, carers and prescribers is needed to better understand the magnitude of these burdens and guide future paediatric formulation development.

## Data Availability

The datasets presented in this article are not readily available because of Ethics Committee restrictions. Requests to access the datasets should be directed to DH, dhawcutt@liverpool.ac.uk (Corresponding author).
